# Understanding Doping, Vacancy, Lattice Stability, and Superconductivity in K*_x_*Fe_2−_ *_y_*Se_2_

**DOI:** 10.1002/advs.201600098

**Published:** 2016-05-17

**Authors:** Yu Liu, Gang Wang, Tianping Ying, Xiaofang Lai, Shifeng Jin, Ning Liu, Jiangping Hu, Xiaolong Chen

**Affiliations:** ^1^ Research and Development Center for Functional CrystalsBeijing National Laboratory for Condensed Matter PhysicsInstitute of PhysicsChinese Academy of SciencesBeijing100190P.R. China; ^2^ Beijing National Laboratory for Condensed Matter PhysicsInstitute of PhysicsChinese Academy of SciencesBeijing100190P.R. China; ^3^ Collaborative Innovation Center of Quantum MatterBeijingP.R. China

**Keywords:** density functional calculations, metal‐intercalated iron selenides, phase diagrams, superconductors

## Abstract

**Metal‐intercalated iron selenides** are a class of superconductors that have received much attention but are less understood in comparison with their FeAs‐based counterparts. Here, the controversial issues such as Fe vacancy, the real phase responsible for superconductivity, and lattice stability have been addressed based on first‐principles calculations. New insights into the distinct features in terms of carrier doping have been revealed.

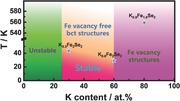

The discovery of superconductivity in A*_x_*Fe_2−_ *_y_*Se_2_ (A = K, Cs, Rb, Tl/Rb, Tl/K)^1^, ^2^, ^3^, ^4^, ^5^, ^6^ triggered another surge of research on iron‐based superconductors, which were previously only featured by iron pnictides^7^, ^8^, ^9^, ^10^, ^11^, ^12^ and β‐FeSe.^13^ The new series of superconductors can be regarded as the formation from the intercalation of metals between the FeSe layers of β‐FeSe. In comparison to the iron pnictides, metal‐intercalated iron selenides are much more complicated in terms of structure, chemical composition, and phases. The nature of the superconducting (SC) phase, for example, is still in debate though considerable progress has been made over the last few years.^14^, ^15^, ^16^

One of the most controversial issues is whether the SC phase is Fe vacancy free or not, i.e., the FeSe layers in A*_x_*Fe_2−_ *_y_*Se_2_ are stoichiometric (*y* = 0), or off‐stoichiometric due to the existence of considerable Fe vacancies. The origin of this issue is largely due to the phase separation that inevitably occurs in these systems at 500–578 K,^17^ leading to the coexistence of the insulating phase (A_2_Fe_4_Se_5_) and the SC phase.^18^, ^19^, ^20^, ^21^, ^22^, ^23^ The thus‐obtained SC phase is not standing freely; instead, it intergrows with the insulating phase in the form of nanostrip and its volume fraction is quite low, 10%–20%, as estimated by various measurements.^24^, ^25^, ^26^, ^27^, ^28^, ^29^ This is the main obstacle that prohibits the precise determination of the structure and the composition of the SC phase. Ying et al.^30^, ^31^ verified that the SC phases in the K‐intercalated iron selenides are almost no Fe vacancy in the FeSe layers based on their study of the superconductors obtained via a liquid ammonia route. The neutron diffraction showed that the 43 K SC phase in Li/ammonia co‐intercalated FeSe compound is Fe vacancy free in a separate study.^32^ Guo et al.^33^ synthesized a 37 K SC phase containing slight Fe vacancy by the similar liquid ammonia method. In the K*_x_*Fe_2−_ *_y_*Se_2_ film grown on SrTiO_3_ (001) substrate, an Fe‐vacancy‐free phase KFe_2_Se_2_ with √2 × √5 charge ordering was observed in the SC region.^34^, ^35^ Different opinions, however, exist. For example, the SC phases are thought to originate from superstructures due to Fe vacancy, such as 2 × 4^36^ or √8 × √10.^37^ Meanwhile, phases with disordered Fe vacancies were also reported to be SC.^38^, ^39^ Apart from Fe vacancies, the possibility of the existence of about 20% excess Fe in an SC phase with transition temperature (*T*_c_) of 44 K in the K‐Fe‐Se system was also reported.^40^

Another puzzling issue is the existence of several SC phases with different *T*_c_ from 30 to 46 K, which are often mixed in a single sample. For instance, apart from a dominated 30 K phase, a 44 K phase of trace amount sometimes can be observed in a K*_x_*Fe_2−_ *_y_*Se_2_ sample obtained by a high‐temperature route.^41^ The amount of 44 K phase can be enhanced in samples with a little less K content (*x* = 0.6–0.7), but its real compositions are unknown. The results in ref.^31^ revealed that the obtained SC phases differ only in K contents, K_0.3_Fe_2_Se_2_ with a *T*_c_ = 44 K and K_0.6_Fe_2_Se_2_ with a *T*_c_ = 30 K. Superconductivity with *T*_c_ of 30 K and 43 K was also observed in K*_x_*Fe_2−_ *_y_*Se_2_ samples with √2 × √2 × 1 superstructure due to K vacancy ordering, but the phases for different *T*_c_ have not been specified.^25^, ^28^, ^42^, ^43^ In addition, K‐intercalated iron selenide with excess Fe was also proposed to have *T*_c_ of 44 K.^40^

So far, there has been no consensus on the answers to the above issues. Moreover, the underlying mechanism of the lattice stability and vacancy is also poorly understood, in particular, in terms of the origin for multifarious structures. To address these issues, here, we first study the energetic change and structural evolution as a function of intercalated metal content by taking K*_x_*Fe_2−_ *_y_*Se_2_ as an example using first‐principles calculations. Two competing factors are found to dominate the formation of the phases and the structural evolution. One is due to the energy increase caused by the accumulation of the negative charge in FeSe layers. The other is due to the Coulomb attraction between the K ion layers and the negatively charged FeSe layers. Then we show that the intercalated K content at 0.25 ≤ *x* ≤ 0.6 can stabilize the body centered tetragonal (bct) lattice and Fe is favored to fully occupy its site. At *x* > 0.6, the structure is stabilized by creating Fe vacancies in FeSe layers. At low intercalated K level, *x* < 0.25, the structure will collapse due to the lattice instability. Then a schematic phase diagram is constructed accordingly whereby we speculate the SC phases are K*_x_*Fe_2−_ *_y_*Se_2_ with 0.25 ≤ *x* ≤ 0.6 and *y* = 0. Our finding sheds some light on understanding this distinct SC family and should also be applicable to other metal‐intercalated iron selenides besides K*_x_*Fe_2−_ *_y_*Se_2_.

To begin with, we study the formation energy for the process of K intercalation. The process can be expressed as a chemical reaction

$$xK+2\mathrm{FeSe}\to K_{x}{\mathrm{Fe}}_{2}{\mathrm{Se}}_{2}\left(0<x\leq 1\right)$$

The formation energy per unit cell can be described as Δ*E*_I_ = *E*_K_ *_x_*_Fe2Se2_ −*E*_K0Fe2Se2_ − x*E*_K_, where *E*_K_ *_x_*_Fe2Se2_ and *E*_K0Fe2Se2_ are the total energies of K*_x_*Fe_2_Se_2_ and an assumed K_0_Fe_2_Se_2_ with a similar bct structure but without any K ion, and *E*_K_ is the energy of elemental K. The variation of Δ*E*_I_ versus K content is shown in **Figure** **1**, which indicates that the intercalation of K into FeSe layers is always energetically favored for the bct structure.

**Figure 1:**
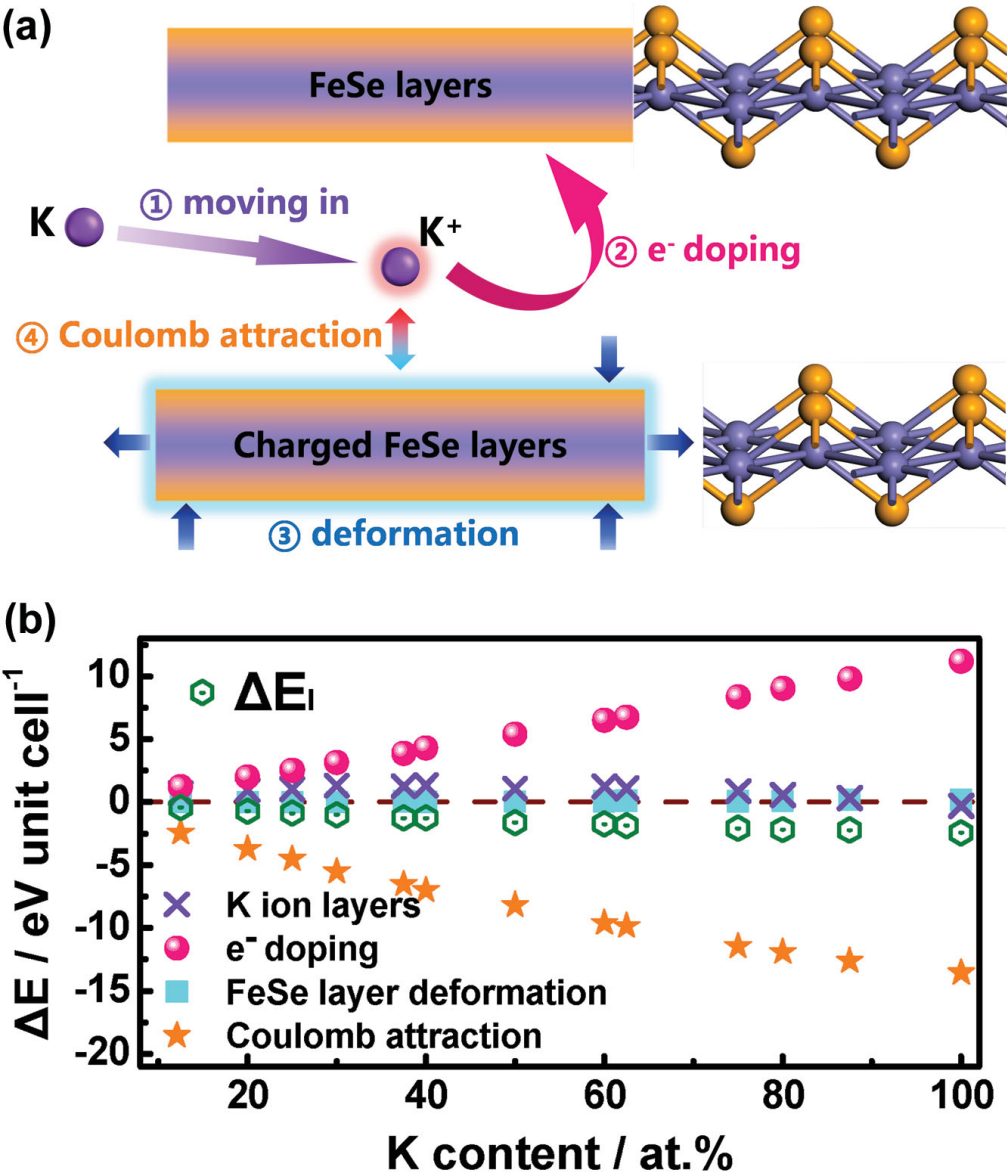
a) The schematic of the K intercalation process. It does not represent a certain structure. b) The total formation energy and the energy change due to the formation of K ion layers, the electron doping in FeSe layers, the deformation of FeSe layers, and the Coulomb attraction between K ion layers and FeSe layers as a function of the K content, respectively.

Then we break down Δ*E*_I_ in terms of energy from the structural constituent units and their interactions. For the K intercalation process, as shown in Figure 1a, K atoms lose their valence electrons and form K ion layers between adjacent FeSe layers after K entering into the lattice, while FeSe layers are charged and deformed and eventually lead to K*_x_*Fe_2_Se_2_ with a bct structure. Hence, the following contributions to the total formation energy are considered:

(1) The formation of K ion layers: Δ*E*_K ion layers_ = *E*_(K ion layers)_ *_x_*_+_ − x*E*_K_
(2) The electron doping in FeSe layers: Δ*E*_e‐ doping_ =*E*_(FeSe layers)_ *_x−_* −*E*_FeSe layers_
(3) The deformation of FeSe layers: Δ*E*_FeSe layer deformation_ = *E*_FeSe layers_ −*E*_K0Fe2Se2_
(4) The Coulomb attraction between K ion layers and FeSe layers: Δ*E*_C_ =*E*_K_ *_x_*_Fe2Se2_ −*E*_(FeSe layers)_ *_x−_* − *E*_(K ion layers)_ *_x_*_+_

And *E*_(K ion layers)_ *_x_*_+_ is the energy of K ion layers, *E*_(FeSe layers)_ *_x_*_−_ the energy of charged FeSe layers, and *E*_FeSe layers_ the energy of FeSe layers in K*_x_*Fe_2_Se_2_. Therefore, the total formation energy can be written as the sum of these four energy changes: Δ*E*_I_ = Δ*E*_K ion layers_ + Δ*E*_e‐doping_ + Δ*E*_FeSe layer deformation_ + Δ*E*_C_.

The variations of these four energy contributions as a function of the K content are calculated and the results are shown in Figure 1b, respectively. First of all, FeSe layers are prominent in contributing more and more positive energy to Δ*e*_I_ when they are negatively charged with more and more electrons. This is easily understood since charging a neutral FeSe sheet will incur additional energy like charging a capacitor. Inversely, with the increasing K content, more charges will come into effect in the Coulomb attraction between K ion layers and the charged FeSe layers and thus greatly enhance |Δ*E*_C_|, which will sufficiently offset the increase of energy induced by electron doping into the FeSe layer. In contrast with these two contributions, the other two energy contributions due to the formation of K ion layers and the deformation of FeSe layers are much smaller. Therefore, we conclude that the former two contributions dominate the energy change during the K intercalation.

To understand the tendency for appearance of Fe vacancy in the K‐intercalated iron selenides, we go further to consider the energy change Δ*E*_Fe vacancy_ (= Δ*E*_I_′ − Δ*E*_I_ −*μ*_Fe_, where Δ*E*_I_′ = *E*_K_ *_x_*_Fe1.94Se2_ − *E*_K0Fe1.94Se2_ − x*E*_K_ is the formation energy per unit cell in the Fe deficiency structure and *μ*_Fe_ is the Fe chemical potential.) by removal of an Fe atom from the unit lattice of K*_x_*Fe_2_Se_2_ and its dependence on the K content. It is found that Δ*E*_Fe vacancy_ only fluctuates above and below zero when *x* ≤ 0.6 as shown in **Figure** **2**a. When *x* > 0.6, Δ*E*_Fe vacancy_ rapidly drops, revealing that Fe vacancies are favored at high levels of K intercalation. This is easily understood since K has a smaller electronegativity than Fe, when the doped electrons due to K are over a limit that Fe—Se bond can accommodate, the surplus electrons will prefer to transfer into Fe^2+^. In this way, Fe is repelled out of the lattice in the form of an element. The final compound with Fe vacancy is more energetically a favored state. The limit is around at *x* = 0.6. The K_2_Fe_4_Se_5_ phase contains 20% Fe vacancy corresponding to *x* = 0.8 is a manifestation of this argument. This tendency of appearance of Fe vacancy with K content is supported by the experimental results.^30^, ^31^, ^32^

**Figure 2:**
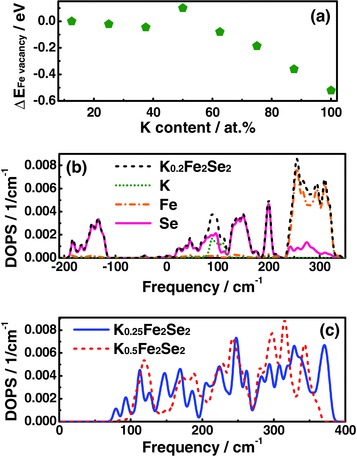
a) The relative variation of formation energy change between K*_x_*Fe_1.94_Se_2_ and K*_x_*Fe_2_Se_2_ as a function of the K content. As the absolute value will be determined by Fe chemical potential (Δ*E*_Fe vacancy_ = Δ*E*_I_′ − Δ*E*_I_ −*μ*_Fe_), the change of formation energy between K_0.125_Fe_1.94_Se_2_ and K_0.125_Fe_2_Se_2_ is set to zero to show the relative values for comparison. b) Total and partial DOPS of K_0.2_Fe_2_Se_2_. c) DOPS of K_0.25_Fe_2_Se_2_ and K_0.5_Fe_2_Se_2_.

Besides the above considerations on the formation energy, the influence of lattice dynamics on the structural stability should also be accounted for. This is, in particular, true for the compounds with low levels of intercalated K, which can induce lattice instability. Figure 2b shows the density of phonon states (DOPS) of K_0.2_Fe_2_Se_2_, where negative frequencies appear, meaning the structure is unstable. Partial DOPS further indicate that it is mainly because of the considerable amount of highly unstable Se atoms, which are unbonded due to the absence of K nearby. The phonon density, however, exhibits no imaginary mode for all other compounds with *x* ≥ 0.25. As examples, Figure 2c shows the DOPS for K_0.25_Fe_2_Se_2_ and K_0.5_Fe_2_Se_2_. The above results demonstrate that the phases of K*_x_*Fe_2_Se_2_ free of Fe vacancy are both energetically and dynamically favored in the K intercalation range of 0.25 ≤*x* ≤ 0.6.

Now we are focused on the trend of structural change upon K intercalation in the range of 0.25 ≤*x* ≤ 0.6. Both electronic and size effects of K will be taken into account. In order to better understand the role of electron doping in the structural change, we charge K_0_Fe_2_Se_2_ with various electron concentrations, which allows us to explore the effect of electron doping while excluding the size influence of K. As shown in **Figure** **3**a,b, the changes in the lattice constant *a*, the Fe—Se bond, and the Se—Fe—Se angle clearly indicate that FeSe layers are stretched along the *ab* plane upon electron doping alone. (Note the results of the highly electron‐doped are extrapolated from the trend of the positively charged ones simply because the stable negatively charged K_0_Fe_2_Se_2_ cannot always be obtained during the iterative calculations.) For a more realistic case, other factors such as the lattice mismatch and the Coulomb attraction must be included. Figure 3c shows the lattice parameter variations with K intercalation, which accounts for all these contributions together with the electron doping. We see that the overall effect of K intercalation increases the lattice constant *a*, stretches FeSe layers, and reduces the lattice constant *c*. For instance, the lattice constant *a* of K_0.25_Fe_2_Se_2_ and K_0.5_Fe_2_Se_2_ expands from 3.65 Å to 3.69 Å and *c* shrinks from 14.42 Å to 13.92 Å. The predicted lattice constant *c* of K_0.25_Fe_2_Se_2_, 14.42 Å agrees with 14.28(4) Å of K_0.3_Fe_2_Se_2_ having *T*_c_ of 44 K.^31^ And the predicted lattice constants *a* and *c* for K_0.5_Fe_2_Se_2_, 3.69 Å and 13.92 Å are consistent with the periods observed using scanning tunneling microscope along [110] and [001], 5.5 Å (√2a) and 14.1 Å,^26^ respectively, considering the calculation accuracy. It is worth noting that a similar trend of the changes in lattice parameters was observed in other electron‐doped ThCr_2_Si_2_ structures, such as KFe_2_As_2_ 9, 44 and CaFe_2_As_2._ 45

**Figure 3:**
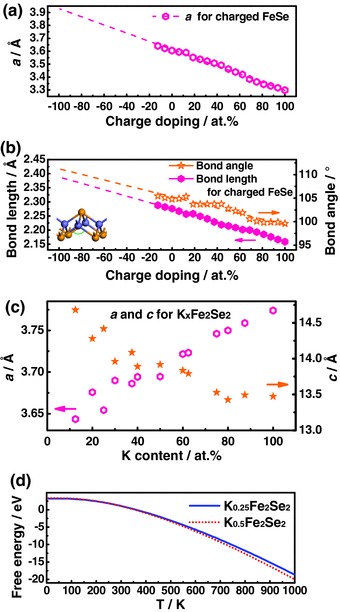
a) The lattice constant *a*, b) the Fe—Se bond length and the Se—Fe—Se bond angle (see details in the inset) along FeSe layers as a function of charge per unit cell. Positive charge means hole‐doped and negative one being electron‐doped. Dashed lines represent the results of linear fitting. c) The variation of the lattice constants *a* (pink rings) and *c* (orange stars) with the K content in K*_x_*Fe_2_Se_2_. d) The free energy of K_0.25_Fe_2_Se_2_ and K_0.5_Fe_2_Se_2_ as a function of temperature.

Furthermore, we explore the temperature‐dependent stability of structures in the K region of interest. To this end, we perform the molecular dynamics (MD) simulations on K_0.25_Fe_2_Se_2_ and K_0.5_Fe_2_Se_2_. Both structures can survive for at least 1 picosecond using 2√2 × 2√2 × 1 super cells at temperatures up to 500 K, indicating that they are stable at these temperatures.^46^ It should be worth noting that the atom displacements of K_0.25_Fe_2_Se_2_ are larger than those of K_0.5_Fe_2_Se_2_, suggesting that K_0.25_Fe_2_Se_2_ is less stable. The variations of free energy with temperature for the two phases are also calculated and shown in Figure 3d. The free energy is more favorable for K_0.5_Fe_2_Se_2_ at temperatures above 268 K, consistent with the result obtained by MD. Despite their variations in stability, both of them have the electronic structures similar to KFe_2_Se_2_ (see Figure S3, Supporting Information), which suggests that they should have similar properties to KFe_2_Se_2_. Considering stoichiometry, formation energy, stability, and electronic structures, K_0.25_Fe_2_Se_2_ and K_0.5_Fe_2_Se_2_ (see Figure S2, Supporting Information for their structures) are proposed to be responsible for the observed superconductivity at 44 and 30 K,^31^ respectively. The relative stability difference could also explain the difficulty to obtain the 44 K phase.

Based on the results presented above, we schematize a phase diagram for the K*_x_*Fe_2–_ *_y_*Se_2_ system in **Figure** **4**. In the K‐rich portion with *x* > 0.6, phases with Fe vacancies tend to exist, agreeing well with the observed antiferromagnetic K_2_Fe_4_Se_5_ phase. As for the low level K‐intercalated compounds (*x* < 0.25), there has been no report on the synthesis of free‐standing K*_x_*Fe_2_Se_2_. We note that the two identified SC phases K_0.3_Fe_2_Se_2_ and K_0.6_Fe_2_Se_2_ lie in the region of 0.25 ≤ *x* ≤ 0.6.^31^ *T*_c_ is 44 K for the former phase and 30 K for the latter one, suggesting that *T*_c_ is dependent on the K content or the doped electron concentrations. Although experimentally observed K contents in SC phases by far are discrete, recent reports about carrier concentration tuning of *T*_c_ from 30 to above 40 K in FeSe thin flakes^47^, ^48^ implies that their variation of *T*_c_ with carrier concentration can be continuous, similar to the cases in other high‐temperature superconductors. Further improvement of *T*_c_ can be expected considering the experimental progresses achieved in single‐layer FeSe films.^49^, ^50^, ^51^, ^52^, ^53^, ^54^ Therefore, we infer that the 30 K phase is electron overdoped and the 44 K phase also might not be optimally tuned. Moreover, in alkali‐metal‐intercalated FeSe compounds prepared at low temperatures, the synergic effect of NH_3_, NH_2_, or C_2_H_4_(NH_2_)_2_ along with alkali metal can stabilize the structures.^31^, ^32^, ^55^ These offer us an effective strategy to raise *T*_c_ of bulk iron‐selenide‐based superconductors by controlling carrier doping while stabilizing the structures by intercalations or effect of substrates. At the moment, tremendous efforts are needed toward this goal. Because of the calculation limit and accuracy, we do not consider the slight off‐stoichiometric cases, say, the Fe vacancy concentration is less than 3 at%. It should be pointed out that such slight off‐stoichiometry is tolerated in K*_x_*Fe_2_Se_2_ just like many other materials which may be the reason that superconductivity was observed in previous reports of refs. 33, 40.

**Figure 4:**
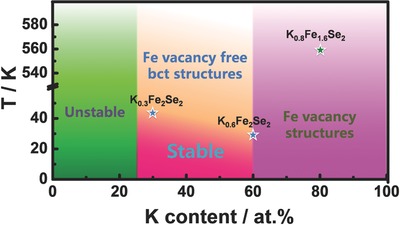
Schematic phase diagram of K*_x_*Fe_2−_ *_y_*Se_2_.

In conclusion, we carefully investigate the energetic change and structural evolution of K*_x_*Fe_2–_ *_y_*Se_2_ as a function of intercalated K content using first‐principles calculations. Two factors dominating the formation of the phases and the structural evolution are confirmed. One is due to the accumulation of negative charge in FeSe layers, the other is due to Coulomb attraction between K ion layers and negatively charged FeSe layers. The structural evolution of this series of phases is summarized: at 0.25 ≤ *x* ≤ 0.6, the bct lattice is stable and Fe is favored to full occupy its site; at *x* > 0.6, FeSe layers tend to exclude Fe atoms and create Fe vacancies; and at *x* < 0.25, the structure will collapse for the dynamic instability. A schematic phase diagram is constructed accordingly and the possible route to further improve *T*_c_ is suggested. The phases responding to the observed superconductivity are proposed to be K_0.25_Fe_2_Se_2_ and K_0.5_Fe_2_Se_2_ in terms of stoichiometry, formation energy, stability, and electronic structures. Though based on the study of K*_x_*Fe_2−_ *_y_*Se_2_, our results should be meaningful to understand the SC and its related phases in metal‐intercalated iron selenides and other similar SC systems.

## Supporting information

As a service to our authors and readers, this journal provides supporting information supplied by the authors. Such materials are peer reviewed and may be re‐organized for online delivery, but are not copy‐edited or typeset. Technical support issues arising from supporting information (other than missing files) should be addressed to the authors.

Supplementary
