# Supplementary material for: Understanding Doping, Vacancy, Lattice Stability, and Superconductivity in KxFe2− ySe2
Source: Adv Sci (Weinh). 2016 May 17;3(10):1600098. doi: 10.1002/advs.201600098 (PMC5096254; doi:10.1002/advs.201600098)
Supplement: Supplementary file 1 — Supplementary [file ADVS-3-0r-s001.pdf]

Copyright WILEY-VCH Verlag GmbH & Co. KGaA, 69469 Weinheim, Germany, 2016.

ADVANCED SCIENCE
Open Access

Supporting Information

for *Adv. Sci.*, DOI: 10.1002/advs.201600098

Understanding Doping, Vacancy, Lattice Stability, and Superconductivity in $K_xFe_{2\#y}Se_2$

Yu Liu, Gang Wang, Tianping Ying, Xiaofang Lai, Shifeng Jin, Ning Liu, Jiangping Hu, and Xiaolong Chen**

Copyright WILEY-VCH Verlag GmbH & Co. KGaA, 69469 Weinheim, Germany, 2013.

Supporting Information

Understanding doping, vacancy, lattice stability and superconductivity in $K_xFe_{2-y}Se_2$

Yu Liu, Gang Wang, Tianping Ying, Xiaofang Lai, Shifeng Jin, Ning Liu, Jiangping Hu and Xiaolong Chen**

Y. Liu, Prof. G. Wang, T. Ying, X. Lai, S. Jin, N. Liu and Prof. X. Chen
Research & Development Center for Functional Crystals, Beijing National Laboratory for Condensed Matter Physics, Institute of Physics, Chinese Academy of Sciences, Beijing 100190, China
E-mail: gangwang@iphy.ac.cn; chenx29@iphy.ac.cn
Prof. J. Hu
Beijing National Laboratory for Condensed Matter Physics, Institute of Physics, Chinese Academy of Sciences, Beijing 100190, China
Prof. J. Hu and Prof. X. Chen
Collaborative Innovation Center of Quantum Matter, Beijing, China

1. Simplification of $K_xFe_2Se_2$ superstructures

The example about the minor energy difference of superstructures due to different arrangements of K ions along *c* axis is shown in Figure S1(a) and S1(b). The example about supercells with different lattice constants in *ab* plane have higher ΔE_I compared to those with the same lattice constants is shown in Figure S1(c) and S1(d).

2. The proposed structures of $K_xFe_2Se_2$ (x ≈ 0.3 and 0.6)

The possible structures of $K_xFe_2Se_2$ with uniform K distribution are proposed, $K_{0.25}Fe_2Se_2$ and $K_{0.5}Fe_2Se_2$. For x ≈ 0.3, $K_{0.25}Fe_2Se_2$ with K ion layers in a

superstructure of 2 × 2 is proposed and the arrangement of K ions is shown in Figure S2(a). In Figure S2(b), $K_xFe_2Se_2$ (x ≈ 0.6) is with the superstructure √2 × √2 of K ion layers, which is actually with composition of $K_{0.5}Fe_2Se_2$. This may be the structure mentioned in previous reports [1, 2]. ΔE_I of $K_{0.25}Fe_2Se_2$ (-0.87 eV/unit cell) is higher than that of $K_{0.5}Fe_2Se_2$ (-1.62 eV/unit cell), implying that $K_{0.25}Fe_2Se_2$ should be less stable than $K_{0.5}Fe_2Se_2$ at the ground state.

3. The electronic structures of $K_xFe_2Se_2$ (x ≈ 0.3 and 0.6)

Calculations of total and partial density of states (DOS) are performed to investigate the effects of superstructures on the properties. In Figure S3(a) and S3(b), Fe 3*d* and Se 4*p* orbital hybridization is located between -7 to -3 eV, which is responsible for the formation of FeSe layers in both $K_{0.25}Fe_2Se_2$ and $K_{0.5}Fe_2Se_2$. Part of Fe 3*d* orbital is located around the Fermi level and decides the properties of both structures. As shown in Figure S3(c), the main difference among total DOS of $K_{0.25}Fe_2Se_2$, $K_{0.5}Fe_2Se_2$, and KFe_2Se_2 is the locations of their Fermi levels. If the Fermi level of KFe_2Se_2 is set to zero, the values of Fermi levels for $K_{0.25}Fe_2Se_2$ and $K_{0.5}Fe_2Se_2$ are about -260 and -200 meV, respectively. So the properties of $K_{0.25}Fe_2Se_2$ and $K_{0.5}Fe_2Se_2$ should be basically the same with that of KFe_2Se_2, while their carrier concentrations are different so that they may have different transition temperatures in terms of superconductivity.

4. Methods

All the first-principles calculations employ generalized gradient approximation based on density functional theory in the form of Perdew-Burke-Ernzerhof function [3]

for exchange-correlation potential, which are performed in the Cambridge Serial Total Energy Package [4]. The self-consistent field method is used with a tolerance of 5.0 × 10^{-7} eV/atom, which is in conjunction with plane-wave basis sets of cutoff energy of 330 eV and ultrasoft pseudopotentials in reciprocal space [5]. According to Monkhorst-Pack special k-point scheme [6], the first Brillouin zone is sampled with grid spacing of 0.04 $Å^{-1}$. Fully optimization of the atomic positions and lattice parameters of all compounds is done until the remanent Hellmann-Feynman forces on all components are less than 0.01 eV/Å. The finite displacement method is used to calculate density of phonon states. In order to examine the dynamic stability of the structures, we perform first-principles finite temperature molecular dynamics simulations with time steps of 10 femtosecond under pressure of 0.1 MPa. Free energies are calculated as a function of temperature using the quasiharmonic approximation based on phonon dispersion [7].

The initial model of KFe_2Se_2 is isostructural to $BaFe_2As_2$ [8]. Superstructures due to different arrangements of K ions along *c* axis are not considered for the minor energy difference. For example, the difference of ΔE_I between two kinds of 1 × 1 × 2 supercells with composition of $K_{0.5}Fe_2Se_2$ [see Figure S1(a) and S1(b)], is only 0.4 meV/unit cell. Superstructures containing one K layer and one FeSe layer due to different arrangements of K ions perpendicular to *c* axis are the main concerns. The ΔE_I of 4 × 1 × 1 supercell with composition of $K_{0.5}Fe_2Se_2$ [see Figure S1(c)] is 26 meV/unit cell higher than that of 2 × 2 × 1 supercell with the same composition [see Figure S1(d)]. Hence, supercells with different lattice constants in *ab* plane, like 3 × 1

× 1, 3 × 2 × 1 and so on, are not discussed in this work for their higher ΔE_I compared to those with the same lattice constants in *ab* plane. Supercells $\sqrt{2} \times \sqrt{2} \times 1$, $2 \times 2 \times 1$, $\sqrt{5} \times \sqrt{5} \times 1$ and $2\sqrt{2} \times 2\sqrt{2} \times 1$ are built accordingly with uniform K distribution as the Coulomb repulsion between K ions dominates K distribution. To study the formation of Fe vacancy, a $2\sqrt{2} \times 2\sqrt{2} \times 1$ supercell ($a = b > 10.3$ Å and $c > 13.5$ Å), which is large enough to accommodate a single Fe vacancy while keeping the tetragonal symmetry, is therefore chosen. In this case, the Fe content corresponds to y = 0.06. The above structures are only designed to obtain different K contents in $K_xFe_{2-y}Se_2$ (y = 0 or 0.06), which shall not represent the real ones. As K ion ordering cannot guarantee the tetragonal symmetry in the structures, the obtained lattice constants or bond lengths will be the average values. The bond angles are obtained using the average coordinates of Fe and Se atoms. Besides, when we calculate ΔE_I, the total energy of $K_0Fe_2Se_2$ is used instead of that of FeSe so as to offset the additional energy, tens of meV per unit cell induced by the change of cell volume.

References

[1] R. H. Yuan, T. Dong, Y. J. Song, P. Zheng, G. F. Chen, J. P. Hu, J. Q. Li, N. L. Wang, *Sci. Rep.* **2012**, *2*, 221.

[2] A. Ricci, B. Joseph, N. Poccia, G. Campi, N. Saini, A. Bianconi, *J. Supercond. Nov. Magn.* **2014**, *27*, 1003-1007.

[3] J. P. Perdew, K. Burke, M. Ernzerhof, *Phys. Rev. Lett.* **1996**, *77*, 3865-3868.

[4] S. J. Clark, M. D. Segall, C. J. Pickard, P. J. Hasnip, M. I. J. Probert, K. Refson, M. C. Payne, *Z. Kristallogr.* **2005**, *220*, 567-570.

[5] D. Vanderbilt, *Phys. Rev. B* **1990**, *41*, 7892-7895.

[6] H. J. Monkhorst, J. D. Pack, *Phys. Rev. B* **1976**, *13*, 5188-5192.

[7] S. Baroni, S. de Gironcoli, A. Dal Corso, P. Giannozzi, *Rev. Mod. Phys.* **2001**, *73*, 515-562.

[8] M. Rotter, M. Tegel, D. Johrendt, *Phys. Rev. Lett.* **2008**, *101*, 107006.

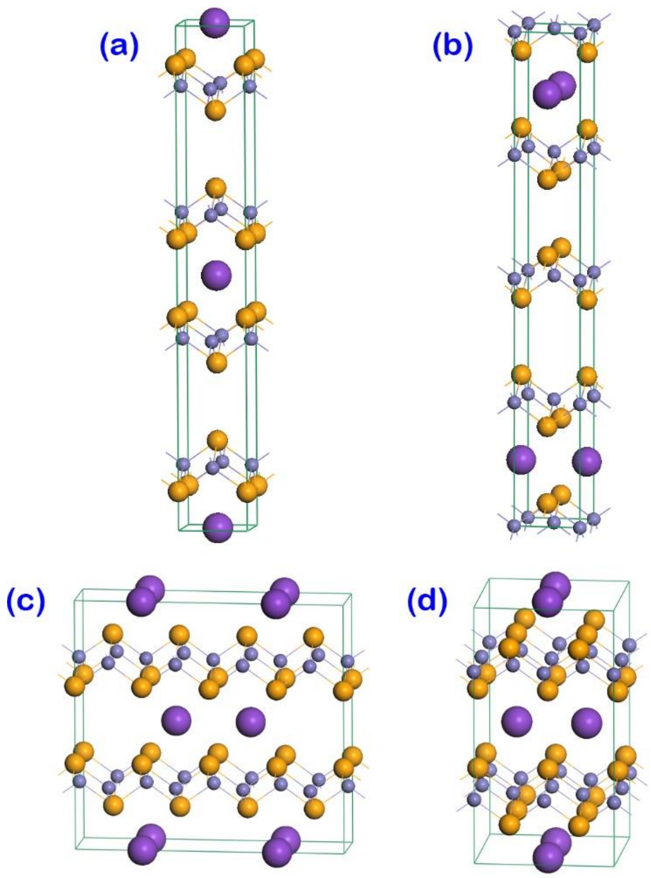

Figure S1 (a) (b) The structures of two kinds of 1 × 1 × 2 supercells with composition of $K_{0.5}Fe_2Se_2$. The former structure always has a vacancy between two K ions while the K ions in the latter appear in pairs along *c* axis. (c) The structure of 4 × 1 × 1 supercell with composition of $K_{0.5}Fe_2Se_2$. (d) The structure of 2 × 2 × 1 supercell with composition of $K_{0.5}Fe_2Se_2$. Violet (largest), grey (smallest) and orange spheres represent K, Fe and Se atoms, respectively.

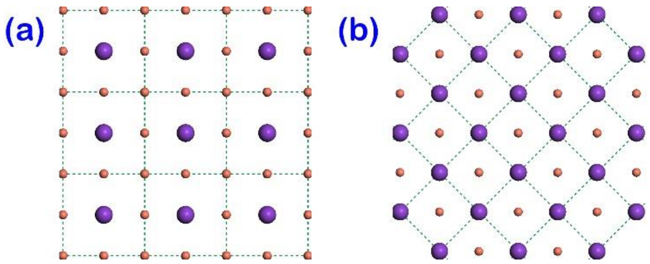

Figure S2 The possible K ion arrangements in $K_{0.25}Fe_2Se_2$ (a) and $K_{0.5}Fe_2Se_2$ (b).

Violet big spheres represent K ions and orange small spheres represent vacancies.

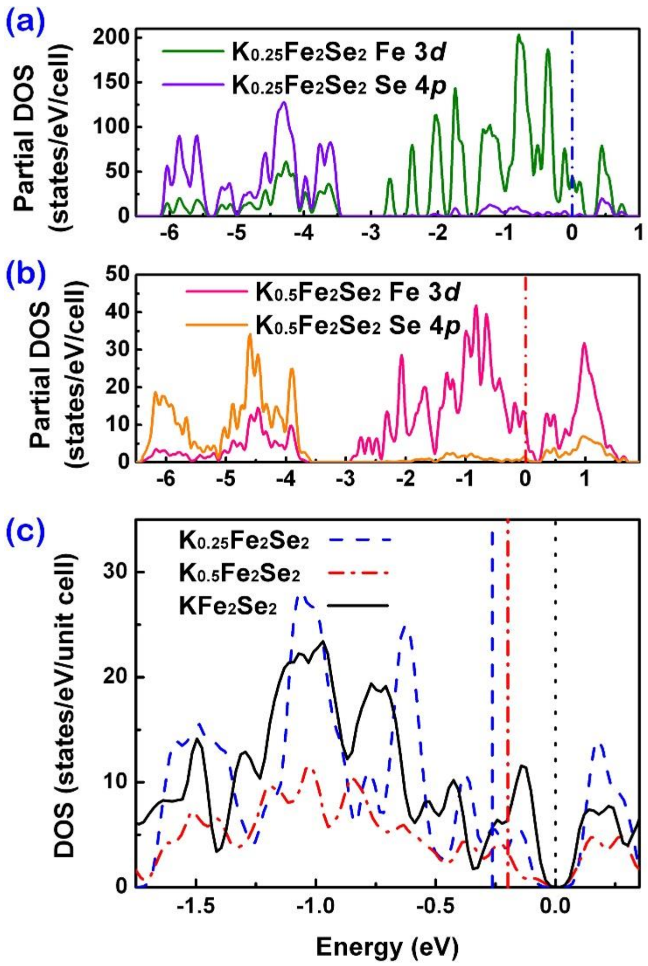

Figure S3 (a) (b) The partial DOS of Fe 3*d* and Se 4*p* in $K_{0.25}Fe_2Se_2$ and $K_{0.5}Fe_2Se_2$. The Fermi level is set to zero. (c) The comparison of total DOS details around the Fermi levels of $K_{0.25}Fe_2Se_2$, $K_{0.5}Fe_2Se_2$ and KFe_2Se_2. The Fermi level of KFe_2Se_2 is set to zero and others are set accordingly.
